# Red Cross first aid training and its effect on public first-aid competency: an empirical study of influencing factors

**DOI:** 10.3389/fpubh.2026.1858371

**Published:** 2026-06-19

**Authors:** Hui Qin, Tao Zeng, Xin Yang

**Affiliations:** 1 Jingchu University of Technology, Jingmen, China; 2 Jingmen Red Cross, Jingmen, China

**Keywords:** first-aid competency, influencing factors, Red Cross, training awareness, training support

## Abstract

**Objective:**

To assess the level of first-aid competency among the public in Jingmen City after receiving Red Cross first aid training and to identify its influencing factors.

**Methods:**

From January to December 2025, a combined approach of offline training, questionnaire surveys, and skill assessments was adopted. A self-designed questionnaire on training awareness and a training support scale were administered to 2,897 residents in Jingmen City. Univariate and ridge regression analysis analyses were conducted to explore factors influencing first-aid competency.

**Results:**

Among 2,964 participants who underwent first-aid training, 2,897 valid questionnaires were collected, yielding a valid response rate of 97.7%. The residents’ training awareness score was 3.79 ± 0.86, training support score was 3.60 ± 0.81, and the overall pass rate for training was 90.02%. The score for first-aid competency was 33.11 ± 6.91. Ridge regression analysis results showed that training awareness, training support, and occupation were influencing factors of first-aid competency (*p* < 0.05).

**Conclusion:**

Following primary Red Cross first aid training, the public demonstrated a moderate level of first-aid competency. However, notable discrepancies were observed, including uneven awareness of first aid importance and inconsistent mastery of knowledge and skills across participants. Future efforts should therefore focus on optimizing training content, instructional methods, and support mechanisms according to the identified influencing factors, with the aim of consolidating and enhancing public first-aid competency.

## 1 Introduction

As the first response measure in public health emergencies and daily accidents, emergency first aid plays an irreplaceable and critical role in saving lives and reducing disability (1). The International Red Cross and Red Crescent Movement has long regarded the dissemination of first aid knowledge as a core mission, promoting “first responder” training as a cornerstone of global first-aid competency systems. In China, implementing first aid training is not only a core statutory responsibility of the Red Cross Society but also a vital practice in supporting the “Healthy China 2030” strategy and building a public-access first-aid competency network.

According to WHO data, injuries and violence lead to approximately 4.4 million deaths globally each year, accounting for nearly 8% of all mortality. Among these, road traffic injuries have become the leading cause of death for individuals aged 5 to 29 (2). In China, the death toll reached 10.93 million in 2024, with 19,626 fatalities resulting from various production safety accidents. Within the 18–34 age group, deaths numbered 654,800 (accounting for 6.0% of total deaths), primarily due to occupational injuries, traffic accidents, and sudden cardiac arrest (3). Furthermore, China’s mortality rates for childhood injuries and drowning remain high, at 8.74 and 3.04 per 100,000, respectively (4). These figures underscore the urgent need for widespread first aid skills among the general public. Training non-professionals in first aid can enhance their first-aid competency, thereby potentially improving health outcomes for individuals facing sudden illness or injury (5).

However, significant challenges persist in promoting first aid knowledge and skills to the broader public (6). Public understanding of first aid and the corresponding ability to act vary considerably, with studies indicating particularly low competency levels among the older adult(s) (7). These challenges may stem from uneven access to training, low skill retention rates, and insufficient public motivation.

Taking the Chinese Red Cross first-aid training model as a starting point, this study explores its impact on the public’s first-aid competency, aiming to provide empirical evidence and policy recommendations for optimizing public first-aid training systems and enhancing the overall level of emergency preparedness in society.

## 2 Methods

### 2.1 Participants

This study employed a cluster sampling method. Between January and December 2025, residents from various counties and districts of Jingmen City were recruited to participate in Red Cross first-aid training sessions and subsequent surveys. The specific clusters were defined as the administrative communities or villages where the training sessions were organized.

Inclusion criteria were: (1) Age 16 years or older; (2) In general good health and physical condition, possessing the basic cognitive and physical abilities necessary to complete the training, including performing practical maneuvers such as kneeling and chest compressions.

Exclusion criteria were: (1) Pregnancy; (2) Self-reported history of severe cardiac conditions (e.g., unstable heart disease) or uncontrolled hypertension; (3) Self-reported severe musculoskeletal issues (e.g., chronic or acute injuries to the back, waist, or knees) that would preclude participation in physical activities.

The study protocol was approved by the Jingmen City Red Cross Society (Approval No: JMHSZH-PX-202401). Prior to participation, all individuals provided written informed consent. Data confidentiality and participant privacy were maintained throughout the research process.

### 2.2 Survey instruments

#### 2.2.1 General demographic data

Demographic data were extracted directly from the official “First Aid at Your Side” Red Cross training management platform (website: www.crcntc.org.cn/spring/login/index). All participants registered on this platform, providing standardized information (e.g., name, gender, education, occupation). Using this existing administrative data ensured completeness, minimized duplicate entry, and reduced participant burden.

#### 2.2.2 First-aid training awareness scale

This 7-item self-report scale assessed participants’ perceptions of first-aid training. Items were developed based on literature, Red Cross guidelines, and expert instructor experience. Content validity was established through a two-round expert review (S-CVI = 0.921). A pilot test (*n* = 150) confirmed good internal consistency (Cronbach’s *α* = 0.889). Items are rated on a 5-point Likert scale (1 = Strongly Disagree, 5 = Strongly Agree); total scores range from 7 to 35, with higher scores indicating greater awareness.

#### 2.2.3 First-aid training support questionnaire

This 12-item questionnaire evaluated perceived training quality across four dimensions: Content Design, Trainer Performance, Organization, and Administration. Items use a 5-point Likert scale (1 = Strongly Disagree, 5 = Strongly Agree); total scores range from 12 to 60. The instrument showed excellent internal consistency (Cronbach’s *α* = 0.895). An open-ended question was included to collect qualitative feedback for program improvement.

#### 2.2.4 First-aid competency assessment

Competency was assessed through self-assessment and objective evaluation. Self-Assessment: A 9-item questionnaire measured perceived mastery of core topics (e.g., CPR, bandaging) on a 5-point scale (1 = Not mastered, 5 = Completely mastered). The total score ranges from 0 to 45, with a higher score indicating stronger first-aid competency.

Objective Evaluation: This consisted of a standardized Red Cross theoretical exam (30 items, 100-point total, pass score ≥80) and a practical skills test covering CPR/AED use and trauma care. Participants had to meet ≥80% of criteria at each skill station to pass.

### 2.3 Data collection and quality control

The study was conducted in collaboration with the Jingmen Red Cross Society. The municipal Red Cross centrally planned the training, with district branches coordinating specifics such as session schedule, venue, participants, and cohort size. Recruitment was announced on the official website 1 week in advance. A dedicated online group was created for each cohort to facilitate communication. Training followed the standard Chinese Red Cross Primary First-Aid Responder manual (modules detailed in Table 1). After training, an online survey link (hosted on Wenjuanxing) was distributed via these groups for uniform data collection. All participants received standardized instructions on the study purpose and confidentiality. To ensure data quality, all survey items were mandatory, and technical restrictions limited submissions to one per IP address to prevent duplicates.

**Table 1:** Standard training schedule for the Chinese Red Cross first responder (basic) certification course.

Training module	Duration (hours)	Training content	Teaching method
Basic knowledge of the Red Cross movement first aid overview	0.5	-Red Cross movement -Emergency first aid and the philosophy of life protection -Purpose, principles, and procedures of first aid	Lecture
Cardiopulmonary resuscitation (CPR) theory	1.0	-Basic knowledge of CPR -Procedures and techniques for adult CPR -Automated external defibrillator(AED)	Lecture demonstration
Foreign body airway obstruction (FBAO)	0.5	Management of foreign body airway obstruction	Lecture demonstration
Trauma care theory	1.0	-Overview of trauma care -Bleeding control and hemostasis methods -Bandaging techniques -Management of ankle sprains -Fracture splinting -Management of burns and scalds -Management of special trauma scenarios	Lecture demonstration
Written examination	0.5	Written examination	
Trauma care skills practice and assessment	1.5	-Hemostasis methods -Bandaging techniques -Fracture splinting -Management of ankle sprains	Demonstration Practice Simulation assessment
CPR skills practice and assessment	3.0	-Adult CPR procedures and techniques -AED use	Demonstration Practice Assessment

One “academic hour” corresponds to one standard teaching hour (approximately 60 min). AED, automated external defibrillator; CPR, cardiopulmonary resuscitation; FBAO, foreign body airway obstruction.

### 2.4 Data analysis

After data cleaning in Excel, analyses were performed using SPSS 26.0. Categorical and continuous variables are presented as frequencies (percentages) and mean ± SD, respectively. Group comparisons used t-tests (two groups) or one-way ANOVA (≥3 groups). Associations were assessed with Pearson’s correlation. Independent influencing factors were identified via multiple linear regression. Statistical significance was set at *p* < 0.05.

## 3 Results

### 3.1 General demographic characteristics

**Figure 1:**
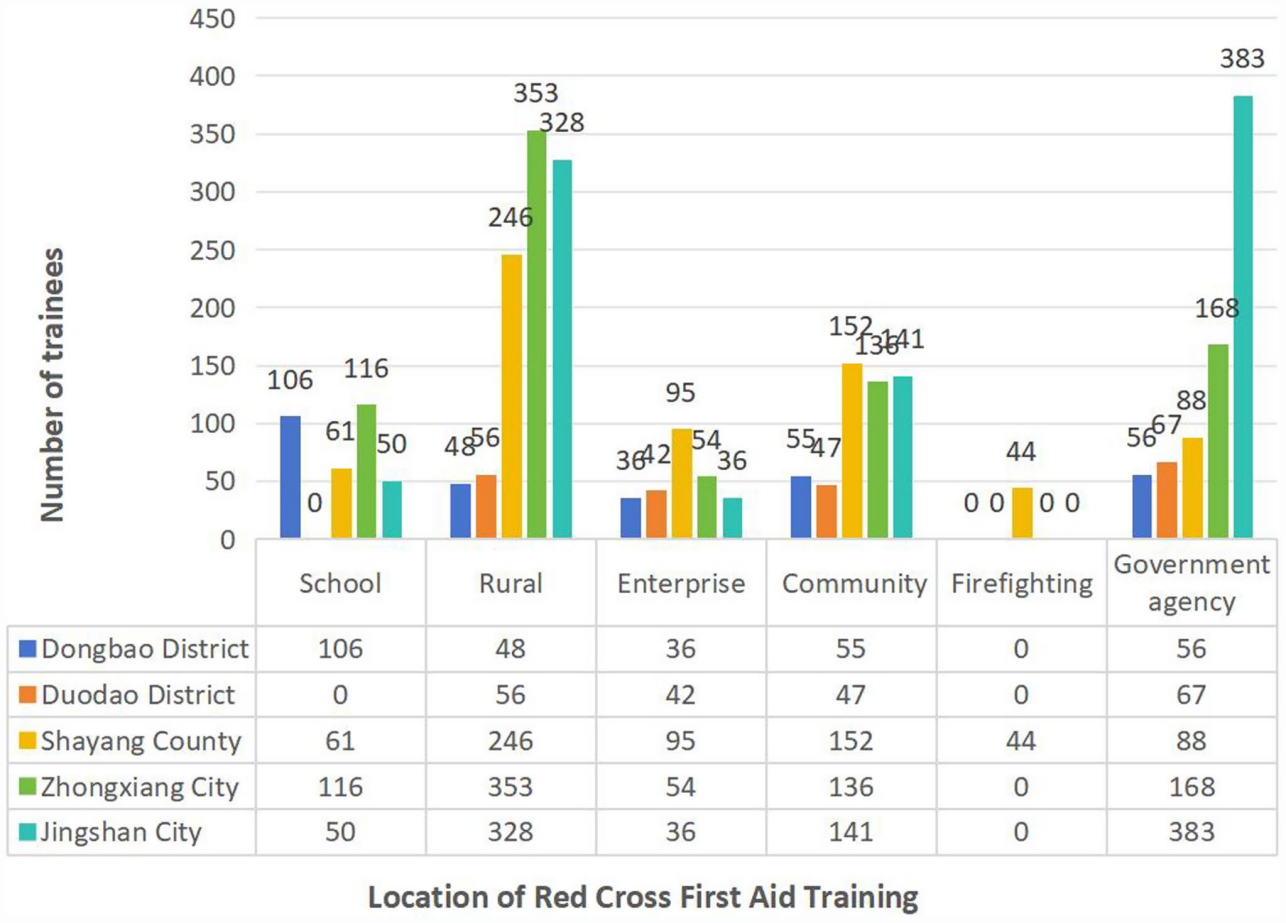
Statistical Table of Red Cross First Aider Training in Jingmen City. Figure shows the total number of participants in Red Cross First Aider training (2,964 persons), without excluding those who failed the questionnaire assessment. (Since the training was conducted under real-name registration while the questionnaire survey was anonymous, the basic information of the excluded individuals could not be identified).

A total of 57 training sessions were conducted, enrolling 2,964 participants across various settings in Jingmen City (Figure 1). The distribution included 8 school-based sessions (*n* = 333), 14 government agency-based sessions (*n* = 762), 19 rural community-based sessions (*n* = 1,031), 4 enterprise-based sessions (*n* = 263), 11 urban community-based sessions (*n* = 531), and 1 session for fire service personnel (*n* = 44). Among the participants, 2,897 individuals completed the study questionnaire, yielding a valid response rate of 97.7%. A total of 67 participants (2.3%) were excluded from the analysis, primarily due to incomplete questionnaire responses or refusal to participate in the survey, with older adult(s) participants accounting for a substantial proportion of this group as they reported difficulty understanding or completing the questionnaire independently. The study population comprised 1,585 males and 1,312 females, with ages ranging from 17 to 70 years. Detailed demographic characteristics are presented in Table 2.

**Table 2:** Univariate analysis of demographic characteristics and training awareness (*n* = 2,897).

Variable	Category	*n* (%)	Training awareness (mean ± SD)	*t*/*F*	*p*-value
Sex	Male	1,585 (54.71)	3.81 ± 0.84	1.492	0.067
	Female	1,312 (45.28)	3.77 ± 0.87		
Age	17 ~ 19	312 (10.77)	3.83 ± 0.60	39.362	<0.001
	20 ~ 29	399 (13.78)	4.11 ± 0.55		
	30 ~ 39	771 (26.62)	3.91 ± 0.79		
	40 ~ 49	715 (24.68)	3.80 ± 0.88		
	50 ~ 59	634 (21.89)	3.47 ± 1.02		
	60 ~ 70	66 (2.27)	3.27 ± 1.06		
Education level	No formal education	219 (7.56)	2.26 ± 0.31	592.026	<0.001
	Primary to high school	792 (27.35)	3.23 ± 1.06		
	Associate degree	1,006 (34.74)	4.11 ± 0.43		
	Bachelor’s degree	852 (29.42)	4.23 ± 0.29		
	Master’s degree or above	28 (0.97)	4.32 ± 0.32		
Occupation	Security and firefighting personnel	44 (1.52)	4.03 ± 0.60	18.707	<0.001
	Teaching personnel	278 (9.60)	4.20 ± 0.35		
	Social and life service personnel	118 (4.07)	3.88 ± 0.82		
	Production personnel	128 (4.42)	3.52 ± 1.01		
	Health professionals	140 (4.83)	4.16 ± 0.52		
	Administrative managers	993 (34.29)	3.75 ± 0.90		
	Students	333 (11.50)	3.89 ± 0.55		
	Transportation service personnel	14 (0.48)	3.85 ± 0.89		
	Retired or unemployed	849 (29.32)	3.63 ± 0.97		
Willingness to volunteer	Yes	2,466 (85.15)	3.80 ± 0.86	0.357	0.361
	No	431 (14.88)	3.78 ± 0.84		

### 3.2 First-aid training awareness score

The mean score for first-aid training awareness among the 2,897 participants was 3.79 ± 0.86, the scores for each item, ranked from highest to lowest, are detailed in Table 3. Awareness scores demonstrated statistically significant differences when compared across groups defined by gender, age, educational attainment, and occupation (*p* < 0.05).

**Table 3:** Item scores of first-aid training awareness (*n* = 2,897).

Item	Score (mean±SD)	Rank
I believe the general public should master basic first-aid knowledge and skills	3.99 ± 0.96	1
I believe first-aid training should be integrated into school curricula or employee onboarding programs	3.94 ± 1.19	2
I believe it is very important to have automated external defibrillators (AEDs) in public places	3.90 ± 1.00	3
l am willing to participate in first-aid training	3.87 ± 1.23	4
I believe refresher training should be conducted every 3 years	3.86 ± 1.06	5
I would voluntarily provide first aid in an emergency	3.82 ± 0.88	6
l am aware of the legal protections for responders under relevant laws in my country	3.18 ± 1.33	7
Total first-aid training awareness score	26.55 ± 5.99	

SD, standard deviation.

### 3.3 First-aid training support

The overall score for perceived training support was 14.38 ± 3.25. Scores across the four measured dimensions, ranked from highest to lowest, were: Training Organization and Implementation (3.68 ± 0.95), Trainer instructor performance (3.65 ± 0.84), Training Content Design (3.56 ± 0.79), and Training Administration (3.49 ± 1.03), the results are presented in Table 4. Pearson correlation analysis revealed significant positive correlations among all these dimension scores (*p* < 0.01).

**Table 4:** Training support scores (*n* = 2,897).

Dimension	Items	Score range	Score (mean ± SD)	Rank
Training content design	3	1–5	3.56 ± 0.79	3
Training instructor performance	3	1–5	3.65 ± 0.84	2
Training organization and implementation	3	2–5	3.68 ± 0.95	1
Training administration	3	1–5	3.49 ± 1.03	4
Overall support	12	7–20	14.38 ± 3.25	

In response to the open-ended question, 38 participants (response rate: 1.31%) provided suggestions for improvement. Key recommendations included: extending hands-on practice time for core skills (e.g., CPR) and incorporating complex scenario simulations; enhancing instructor guidance during practice and integrating multimedia with real-case teaching; optimizing session duration, ensuring adequate training equipment, and providing pre-session materials; and establishing mechanisms for skill retention, such as offering periodic refresher courses, creating an online review platform, and facilitating opportunities for practical application.

### 3.4 First-aid competency scores

In the post-training assessment, 123 participants (4.24%) scored below the 80-point passing threshold on the theoretical exam, and 144 (4.97%) failed the practical skills evaluation. Twenty-two participants (0.76%) did not pass either component. Consequently, the overall training pass rate was 90.02%. Participants’ self-assessed mastery of the training content yielded a mean score of 3.68 ± 0.91(total score 33.11 ± 6.91). Self-rated proficiency was highest for cardiopulmonary resuscitation (CPR) techniques (3.95 ± 1.05) and lowest for foundational Red Cross first-aid knowledge (3.38 ± 0.79) (Table 5).

**Table 5:** First-aid competency scores (*n* = 2,897).

Item	Range	Score (mean±SD)	Rank
Cardiopulmonary resuscitation (CPR)	2–5	3.95 ± 1.05	1
Correctly calling emergency services	1–5	3.91 ± 1.31	2
Bandaging	1–5	3.78 ± 1.24	3
Hemostasis	1–5	3.76 ± 1.13	4
Management of common diseases and conditions	1–5	3.63 ± 1.76	5
Lifting and moving techniques	1–5	3.62 ± 1.09	6
Splinting	1–5	3.61 ± 1.26	7
Proper use of automated external defibrillator (AED)	1–5	3.48 ± 1.18	8
Basic Red Cross first-aid knowledge	1–5	3.38 ± 0.79	9
First-aid competency		33.11 ± 6.91	

SD, standard deviation.

### 3.5 Ridge regression analysis results for first-aid competency scores

Prior to conducting ridge regression, multicollinearity among independent variables was assessed using Pearson correlation coefficients and Variance Inflation Factors (VIF). The correlation matrix revealed an extremely strong positive correlation between “Training Cognition Total Score” and “Training Support” (*r* = 0.983), indicating near-perfect multicollinearity. VIF analysis further confirmed this finding: VIF = 30.13 for Training Cognition Total Score and VIF = 31.21 for Training Support, both substantially exceeding the severe multicollinearity threshold (VIF > 10). All other independent variables had VIF values below 2, indicating no multicollinearity concerns. Given the severe multicollinearity between Training Cognition Total Score and Training Support, Ordinary Least Squares (OLS) regression would produce unstable coefficient estimates and inflated standard errors. Therefore, Ridge Regression was employed as the appropriate analytical approach.

The optimal ridge parameter (*λ*) was determined through 10-fold cross-validation, yielding *λ* = 155.22. The ridge regression model demonstrated good overall fit: *R*^2^ = 0.6334, Adjusted *R*^2^ = 0.6325, indicating that the model explained approximately 63.34% of the variance in Emergency Rescue Ability Total Score. The Root Mean Square Error (RMSE) was 0.4644, representing reasonable prediction accuracy relative to the dependent variable’s standard deviation (SD = 0.7672). The standardized ridge regression coefficient was in Table 6.

**Table 6:** Ridge regression analysis results for first-aid competency scores.

Variable	*β*	SE	*t*	*p*-value	95% CI lower	95% CI upper
Gender	−0.0188	0.0107	−1.764	0.0778	−0.0397	0.0021
Age	−0.0174	0.0105	−1.6573	0.0976	−0.0379	0.0032
Education	0.022	0.0135	1.6285	0.1035	−0.0045	0.0485
Occupation	0.0255	0.011	2.3114	0.0209	0.0039	0.047
Willingness to volunteer	−0.0095	0.0099	−0.964	0.3352	−0.0289	0.0098
Training awareness	0.389	0.0221	17.6111	<0.001	0.3457	0.4323
Training support	0.3749	0.0252	14.8735	<0.001	0.3255	0.4244

*R*^2^ = 0.6334, Adjusted *R*^2^ = 0.6325, *F* = 677.70, *p* < 0.001.

*β*, standardized ridge regression coefficient; SE, bootstrap standard error; CI, confidence interval.

## 4 Discussion

First-aid competency is crucial for public health emergency outcomes, yet global proficiency remains low, with fewer than 30% possessing adequate knowledge and on-site intervention rates below 20% for events like cardiac arrest (8). Consistent with prior studies (9, 10), post-training self-assessed competency in this study was moderate-to-low (33.11 ± 6.91). This may stem from the condensed course format, which prioritizes knowledge delivery over sufficient hands-on practice and iterative feedback, hindering skill consolidation (11, 12). The observed discrepancy between theoretical knowledge, practical performance, and self-ratings further underscores this gap, possibly exacerbated by assessment-related anxiety. Implementing more interactive pedagogical strategies, such as simulation-based training, could enhance practical skill mastery and learner confidence.

Participant awareness of the importance of first-aid training was favorable (3.79 ± 0.86), with support for integrating it into educational and professional onboarding programs (13). Students are a key target group, as trained adolescents can significantly mitigate harm during emergencies (14, 15). However, awareness of legal protections for responders was notably low (3.18 ± 1.33), likely due to insufficient coverage of legal safeguards and risk analysis in current curricula, potentially deterring application of skills in real scenarios (16).

Training support was rated positively overall (14.38 ± 3.25), with organization and implementation scoring highest, attributable to a standardized, well-structured learning experience. Training cognition total score (*β* = 0.3890, *p* < 0.001, 95% CI: 0.3457–0.4323) and training support (*β* = 0.3749, *p* < 0.001, 95% CI: 0.3255–0.4244) are the most important predictors of first-aid competency, with comparable effect sizes and both highly significant. This suggests that in practical training programs, simultaneous attention should be given to enhancing trainees’ cognitive understanding of training and their perceived training support to maximize first-aid competency development (17). Occupation (*β* = 0.0255, *p* = 0.021, 95% CI: 0.0039–0.0470) demonstrated a statistically significant but weak positive effect on emergency rescue ability. Individuals from different occupational backgrounds exhibited differences in emergency rescue ability, though the effect size was minimal. Demographic variables (age, gender, education, occupation) and volunteer willingness showed limited independent predictive power for first-aid competency, further highlighting the central role of training-related factors in capability building. Ridge regression successfully separated the effects of two highly collinear variables, yielding more stable and reliable results. Had OLS regression been used, the multicollinearity-induced instability in coefficient estimates could have led to misleading conclusions.

Synthesizing these findings, a future integrated training model is proposed, combining structured instruction with deliberate practice, scenario simulation, and case-based feedback. This should be coupled with optimized logistics, accessible refresher courses, and digital reinforcement platforms to transition from discrete learning to sustained competency, thereby improving skill retention and real-world preparedness.

## 5 Limitations

While this study utilized a large sample, its generalizability is constrained by several limitations. The sample was recruited from specific areas within Jingmen City and did not employ stratified sampling for key demographic variables such as urban–rural distribution. This may limit the representativeness of the findings for the broader population. Furthermore, the cross-sectional design precludes the establishment of causal relationships between the identified factors and first-aid competency.

## 6 Conclusion

This study concludes that public first-aid competency in Jingmen City is at a moderate level and is significantly influenced by multiple factors, including occupation, training awareness, and perceived training support.

To enhance training effectiveness, it is recommended that Red Cross authorities develop targeted strategies. These should focus on optimizing curriculum design, intensifying hands-on practical training, and improving administrative and logistical support. Crucially, sustaining competency requires moving beyond one-time training. The implementation of regular refresher courses and the establishment of platforms for ongoing practical application are essential to consolidate skills, promote their real-world use, and ultimately strengthen community self-rescue and mutual-aid capabilities.

## Data Availability

The original contributions presented in the study are included in the article/supplementary material, further inquiries can be directed to the corresponding author.
